# Carbapenem-resistant Gram-negative bacteria (CR-GNB) in ICUs: resistance genes, therapeutics, and prevention – a comprehensive review

**DOI:** 10.3389/fpubh.2024.1376513

**Published:** 2024-03-27

**Authors:** Qi Li, Xiaoshi Zhou, Rou Yang, Xiaoyan Shen, Guolin Li, Changji Zhang, Pengfei Li, Shiran Li, Jingxian Xie, Yong Yang

**Affiliations:** ^1^Department of Pharmacy, Sichuan Academy of Medical Sciences & Sichuan Provincial People's Hospital, School of Medicine, University of Electronic Science and Technology of China, Chengdu, China; ^2^Personalized Drug Therapy Key Laboratory of Sichuan Province, School of Medicine, University of Electronic Science and Technology of China, Chengdu, China; ^3^Department of Pharmacy, Chengdu Qingbaijiang District People's Hospital, Chengdu, China; ^4^School of Basic Medicine and Clinical Pharmacy, China Pharmaceutical University, Nanjing, China

**Keywords:** ICU, CR-GNB, mechanism, genes, infection control strategies, predictive model

## Abstract

Intensive care units (ICUs) are specialized environments dedicated to the management of critically ill patients, who are particularly susceptible to drug-resistant bacteria. Among these, carbapenem-resistant Gram-negative bacteria (CR-GNB) pose a significant threat endangering the lives of ICU patients. Carbapenemase production is a key resistance mechanism in CR-GNB, with the transfer of resistance genes contributing to the extensive emergence of antimicrobial resistance (AMR). CR-GNB infections are widespread in ICUs, highlighting an urgent need for prevention and control measures to reduce mortality rates associated with CR-GNB transmission or infection. This review provides an overview of key aspects surrounding CR-GNB within ICUs. We examine the mechanisms of bacterial drug resistance, the resistance genes that frequently occur with CR-GNB infections in ICU, and the therapeutic options against carbapenemase genotypes. Additionally, we highlight crucial preventive measures to impede the transmission and spread of CR-GNB within ICUs, along with reviewing the advances made in the field of clinical predictive modeling research, which hold excellent potential for practical application.

## 1 Introduction

Antibiotics play a vital role in controlling bacterial infections; however, the development of new antibiotics lags far behind the worldwide spread of antimicrobial resistance (AMR) (1, 2). It has been estimated that in 2019, drug-resistant bacterial pathogens were responsible for 1.27 million deaths (3). This number has nearly doubled from the 700,000 deaths reported in 2016 from AMR globally, in just a few years. According to experts, this number could reach 10 million by 2050 if resistance is not reduced or new antibiotics are not developed (4). Carbapenem-resistant Gram-negative bacteria (CR-GNB) possess a high resistance rate against a wide range of antibiotics, further limiting the antibiotic options available for patients. CRE (carbapenem-resistant *Enterobacteriaceae*), CRAB (carbapenem-resistant *Acinetobacter baumannii*), and CRPA (carbapenem-resistant *Pseudomonas aeruginosa*) are classified as pathogens posing a significant threat to human health (4).

Patients admitted to the intensive care unit (ICU) are typically immunocompromised, presenting with multiple comorbidities, overuse of broad-spectrum antibiotics, indwelling catheters, and undergoing multiple invasive procedures, which puts them at a relatively high risk of bacterial infections (5). According to Vincent et al. (6), the incidence of infection in ICU patients exceeds 50%. At present, the commonly employed microbiological methods for diagnosing bacterial infections suffer from a delayed nature, making it challenging to promptly target antibiotics based on drug sensitivity tests. The lack of rapid diagnostic methods to identify resistance genes in the clinical setting, as well as the scarcity of targeted antimicrobials, often results in the overuse of broad-spectrum antibiotics, which is a major contributor to AMR (7). Treatment options for infections caused by CR-GNB are limited and associated with high rates of clinical failure, morbidity, and mortality. Once a CR-GNB infection occurs and is left uncontrolled, it is highly likely to progress into a severe infection and lead to the mortality of patients in the ICU. Understanding the appropriate range of antibiotics for treatment is crucial for establishing an effective treatment strategy initially, alongside enhancing preventive and control measures within the ward to halt pathogen dissemination and deter drug resistance development. This paper offers a comprehensive examination of bacterial resistance mechanisms, CR-GNB resistance genes, and therapeutic options for respective infections. It concludes by outlining strategies for preventing CR-GNB colonization and infections in the ICU, including advancements in infection prediction models for critically ill populations. The application of prediction models in the ICU to promptly identify high-risk groups for CR-GNB infection can provide valuable insights for controlling the spread of CR-GNB in the ICU and improving the prognosis of ICU patients.

## 2 AMR mechanisms

**Figure 1:**
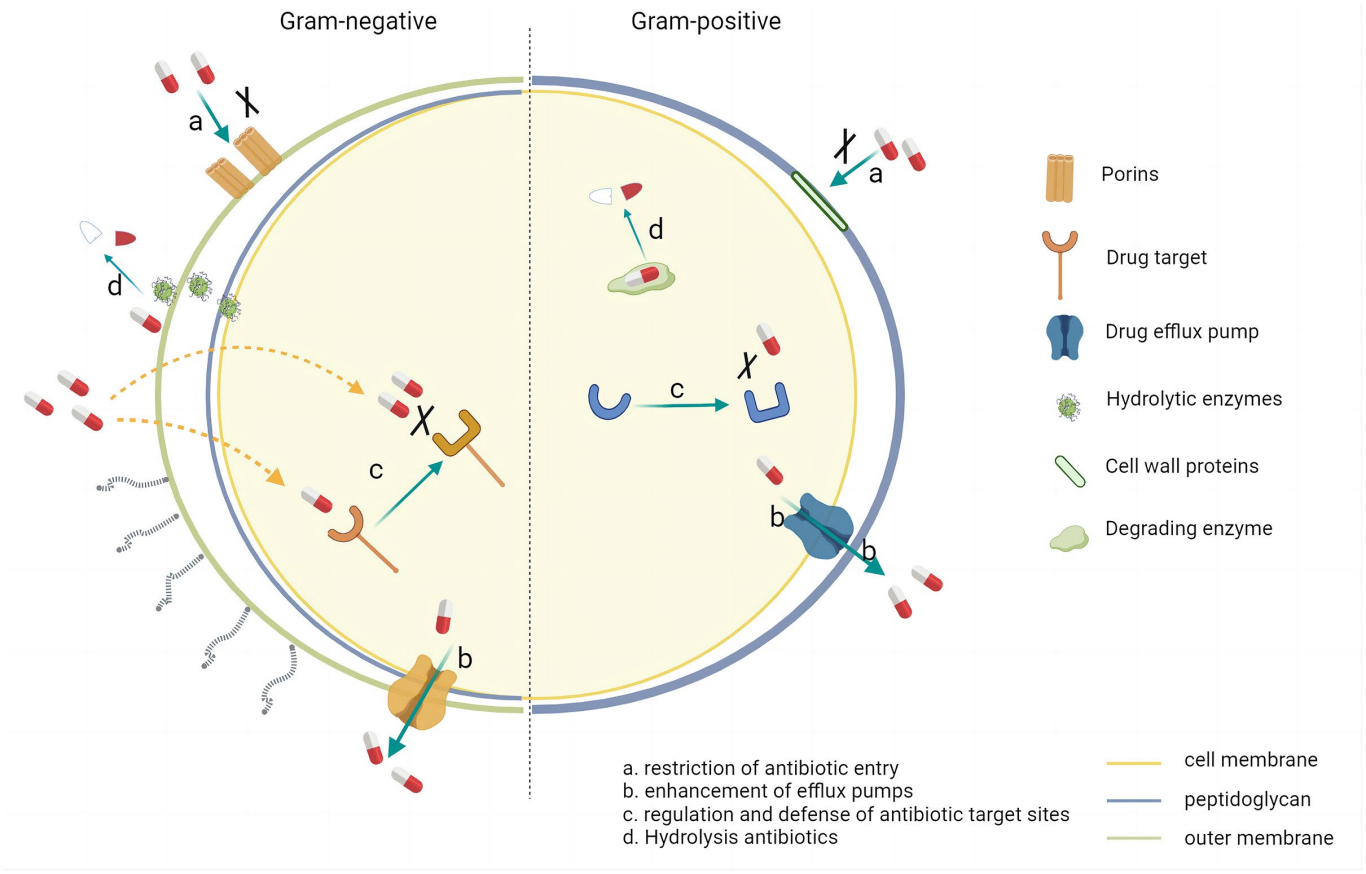
Resistance mechanisms in Gram-negative bacteria and Gram positive bacteria.

The AMR is a complex as well as multifactorial phenomenon. In terms of its mechanism, AMR is associated with both selective pressure on bacteria and horizontal gene transfer between bacteria (8, 9). Figure 1 illustrates complex resistance mechanisms in bacteria: (i) Restriction of antibiotic entry. Many antibiotic targets are within bacteria, reducing the uptake of antimicrobials and thereby preventing their binding to the target site. (ii) Enhancement of efflux pumps. A large amount of antibiotic is released out of the cell, reducing the concentration of antibiotics within the bacteria. (iii) Regulation and defense of antibiotic target sites. Preventing the antibiotic from reaching its binding site and modifying the target site so that the affinity of the antibiotic molecule is reduced. (iv) Production of hydrolytic enzymes. Inactivation of the drug by adding specific chemical parts to the compound or destruction of the molecule itself so that the antibiotic cannot interact with its target. In addition, bacteria can adapt to antibiotic attacks by acquiring key DNA through horizontal gene transfer. Plasmids and transposons play a crucial role in developing and spreading bacterial resistance in clinical infections (10). Many resistance genes are localized on plasmids, and these mobile genetic elements can quickly transfer resistance within or between different bacterial species. Transformation (incorporation of naked DNA), transduction (phage-mediated), and splicing (bacterial “sexing”) are the three main ways. The emergence of resistance in the hospital setting usually involves splicing, a very efficient gene transfer method involving cell-to-cell contact.

**Figure 2:**
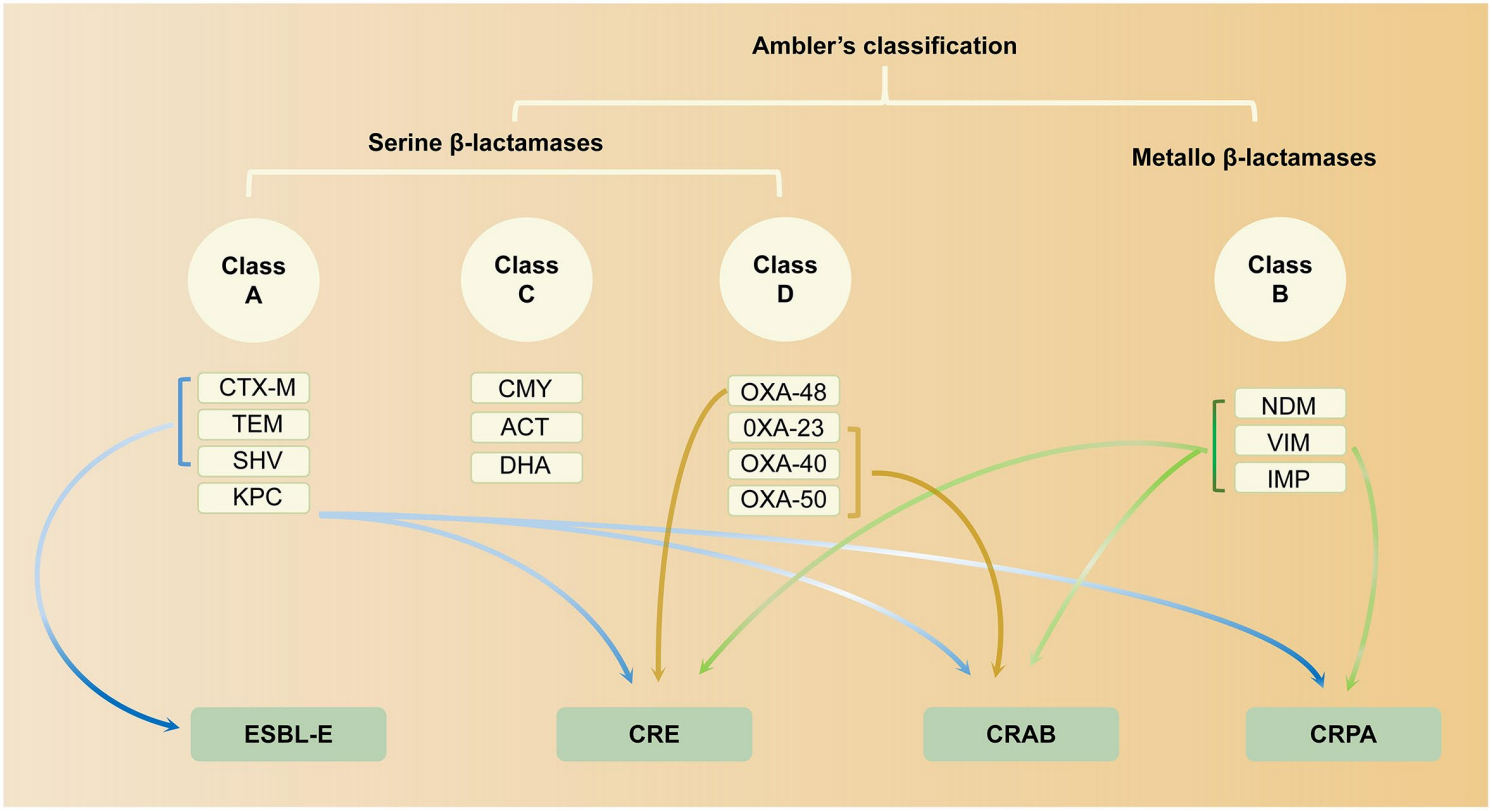
β-lactamases in MDR-GNB according to Ambler’s classification.

The production of β-lactamases is a crucial mechanism of drug resistance in Gram-negative bacteria. Ambler’s classification categorizes these bacteria into four groups: A to D. The enzymes in classes A, C, and D use serine residues in their active catalytic site to hydrolyze β-lactams, while class B enzymes are metallo β-lactamases (MBLs) that contain zinc in their active site (11). Among these, extended spectrum β-lactamases (ESBLs), which belong to Ambler class A, can hydrolyze various β-lactam antibiotics such as cefotaxime, ceftriaxone, and ceftazidime, but they cannot hydrolyze and are resistant to cephalosporins and carbapenems (12). Infections caused by ESBL-producing *Enterobacteriaceae* (EBLS-E), which are mainly *Klebsiella pneumoniae* (*K. pneumoniae*) and *Escherichia coli*, are increasing worldwide (13, 14). The primary resistant genotypes of ESBL include bla_CTX-M_, bla_SHV_, and bla_TEM_ (15). CTX-M type ESBL is the most predominant type of *Enterobacteriaceae* cultured from blood and hydrolyzes cefotaxime and ceftriaxone more effectively than ceftazidime (16). Carbapenem antibiotics, which are atypical β-lactam antibiotics with the broadest antimicrobial spectrum and the strongest antibacterial activity, can prevent cell wall synthesis and lead to bacterial lysis by inhibiting penicillin binding proteins (17). The resistance mechanism of CR-GNB can be classified into enzymatic and non-enzymatic types. Production of carbapenemase is a critical mechanism of GNB resistance to carbapenem antibiotics. The carbapenemase type of CR-GNB is shown in Figure 2. The genes encoding for carbapenemases are highly transmissible and easily spread through plasmid-and transposon-mediated dissemination (18). Non-enzymatic CR is primarily mediated by the acquisition of resistance genes, including mutations in chromosomally encoded porin genes (e.g., OprD) and overexpression of genes encoding efflux pumps (including MexAB-OprM, Mexxy-OprM, and MexCD-OprJ) (19). The genes that lead to resistance to carbapenem antibiotics in different species of Gram-negative bacilli are thus somewhat different. In the following section, the common CR-GNB within the ICU are summarized along with the genes they have been found to cause resistance.

## 3 CR-GNB resistance genes in ICUs

### 3.1 CRE

The Centers for Disease Control and Prevention (CDC) defines CRE as *Enterobacteriaceae* that are resistant to carbapenem antibiotics. In the United States, approximately 13,000 infections caused by CRE have been reported in hospitalized patients, resulting in an estimated 1,100 deaths (20). Patients who require medical devices such as ventilators, urinary catheters, or intravenous catheters, those who are on prolonged antibiotic treatment, and individuals with weakened immune systems are at a relatively high risk of contracting CRE infections (21). Hence, it is crucial to exercise caution in implementing therapeutic measures to avoid unnecessary invasive procedures for patients who usually have underlying medical conditions and those who require ICU-level interventions. Based on the resistance mechanism, CRE can be categorized into carbapenemase-producing enterobacteria (CPE) and non-carbapenemase-producing enterobacteria (non-CPE). CPE comprises carbapenemase-resistant enzymes such as *K. pneumoniae* carbapenemase (KPC) in class A, benzoxacillin carbapenemase/oxacillinase (OXA) in class D, and MBLs belonging to class B, including imipenemase metallo-β-lactamase (IMP), New Delhi metallo-β-lactamase (NDM), and Verona integrase-encoded metallo-β-lactamase (VIM) (11). The bla_KPC_ gene is the most prevalent gene in Ambler class A, and its production plays a significant role in the carbapenem-resistant *K. pneumoniae* (CRKp). On the other hand, bla_OXA-48_ is a more common gene responsible for resistance to carbapenem antibiotics in *Escherichia coli* (22). Single CRE isolate can possess multiple carbapenemase-encoding genes. For instance, in Egypt, where NDM and OXA-48-like enzymes are widespread, polymerase chain reaction results demonstrated that about 90% of *Enterobacteriaceae* isolates harbored one or more carbapenemase-encoding genes, with bla_NDM-1_ being the most prevalent genotype, followed by bla_OXA-48_ (23).

### 3.2 CRAB

*Acinetobacter baumannii* (*A. baumannii*) is the most frequently isolated pathogen in ICUs (24), leading to various infections such as pneumonia, skin and soft tissue infections, and bloodstream infections (BSIs) (25, 26). The emergence of CRAB poses a significant challenge for treatment and has intensified the prevalence of hospital-acquired infections, thus becoming a major threat to global public health (11). The mechanisms of carbapenem resistance in *A. baumannii* involve various factors such as increased efflux pumps, decreased expression or inactivation of pore proteins, modifications of penicillin-binding proteins, and production of several types of β-lactamases (27, 28). The most common mechanism observed in CRAB is the production of carbapenemases, and the genes encoding the acquired carbapenemases play a key role. Among carbapenemases, the OXA enzymes are the most frequently reported in *A. baumannii*, such as OXA-23, OXA-24, OXA-40, OXA-51, OXA-58, and OXA-143 (29, 30). Additionally, metastable MBLs, including VIM, IMP, and NDM enzymes, have also been linked with drug resistance phenotypes in *A. baumannii* (11). It is essential to note that although KPC enzymes have primarily been detected in *K. pneumoniae*, variants of bla_KPC_, such as bla_KPC-2_ and bla_KPC-3_, have been reported in *A. baumannii* in a Brazilian hospital (31). The acquisition of bla_KPC_ might be associated with *A. baumannii*’s resistance to carbapenems.

### 3.3 CRPA

*Pseudomonas aeruginosa* (*P. aeruginosa*) is a Gram-negative bacterium commonly found in moist environments, such as washing tanks, aerators, respirators, and other equipment, as well as solutions exposed in hospital environments (32). It is a significant cause of healthcare-related problems, leading to urinary, respiratory, and BSIs in long-stay hospitalized patients (11, 33). These infections can be fatal in critically ill and immunocompromised patients in ICUs, and they may be further exacerbated by AMR (34). Infections caused by CRPA result in longer hospitalization periods and higher mortality rates compared to infections caused by carbapenem antibiotic-sensitive strains (35, 36). The development of CRPA involves the interaction of several complex resistance mechanisms. Firstly, the upregulation of efflux pumps (e.g., MexAB-oprM) allows for increased drug efflux, leading to resistance against most β-lactams (37). Additionally, the loss of OprD outer membrane proteins, which normally prevent the entry of antibiotics, coupled with the overproduction of Ambler C-like enzymes, can result in the near-exhaustion of *P. aeruginosa*’s resistance to β-lactams (38). Resistance to carbapenem antibiotics through carbapenemase production is a less common mechanism (39). Out of 28 CRPA strains isolated in the ICU, only three strains produced KPC (40). However, carbapenemase production as a resistance mechanism appears to be increasingly common, with bla_VIM_ in MBLs being the most commonly detected gene, typically encoded on plasmids that are highly capable of dissemination (38). In instances where CRPA lacks carbapenemases, resistance is typically due to the absence of OprD or the overexpression of efflux pumps.

## 4 Treatment options

Given the limited therapeutic options for extensively drug-resistant Gram-negative bacteria, it is crucial to adopt a rational approach in utilizing available antibiotics to mitigate the emergence and spread of AMR. To effectively manage infections caused by CR-GNB, it is recommended to carefully select appropriate therapeutic agents based on the genetic characteristics of the bacteria. Below, it provides a concise summary of the mechanisms and efficacy of therapeutic selection. The activity of the treatment options on CR-GNB is summarized in Table 1.

**Table 1:** List of treatment options against carbapenemase-producing Gram-negative organisms.

Treatment options	CPE			CRPA	CRAB
	KPC	MBLs	OXA		
Ceftazidime-avibactam	+	−	+	+^**^	−
Meropenem-vaborbactam	+	−	−	+^*^	−
Imipenem-relebactam	+	−	−	+^*^	−
Cefiderocol	+	+	+	+	+
Polymyxins	+			+	+
Tigecycline/minocycline	+			−	+
Aminoglycosides	+			−	−

+, active; −, not active; ^*^KPC-producing CRPA; ^**^KPC and OXA producing CRPA.

### 4.1 Ceftazidime/avibactam

Avibactam binds reversibly to β-lactamases and exhibits activity against carbapenemases, thereby restoring the inhibitory activity of ceftazidime against the majority of CRE and CRPA. Ceftazidime/avibactam generally demonstrates high efficacy against organisms producing KPC, although resistance has been observed in isolates producing KPC-2 and KPC-3, which may be attributable to reduced porin expression (41). Combination therapy could reduce mortality in BSIs caused by KPC-producing *K. pneumoniae* (42). However, the use of ceftazidime/avibactam in combination with other antimicrobial agents for the treatment of CRE and CRPA infections did not exhibit significant advantages in terms of survival and cure rates (43, 44). Ceftazidime/avibactam alone demonstrates superior effectiveness in patients with OXA-48-producing CRE infections compared to treatment with colistin, tigecycline, and meropenem (45, 46). Therefore, prioritizing ceftazidime/avibactam for the treatment of KPC-producing or OXA-48-producing CRE, as well as CRPA, may improve survival rates among patients in the ICU and reduce the risk of renal injury, as opposed to selecting alternative drugs or multidrug combinations. Moreover, since avibactam does not inhibit MBLs (NDM, VIM, and IMP), combining it with aztreonam, a drug stable against metallo-β-lactamases, may be a potential therapeutic strategy for treating CR-GNB infections belonging to class B. The combination of ceftazidime/avibactam and aztreonam exhibits good *in vitro* activity against *Enterobacteriaceae* producing metallo-β-lactamases, with favorable *in vitro* effectiveness (47).

### 4.2 Meropenem/vaborbactam

Vaborbactam is a β-lactamase inhibitor that primarily targets KPC carbapenemases but not MBLs, as well as class D β-lactamases (48). On the other hand, meropenem effectively treats Gram-negative bacilli, such as *K. pneumoniae*, *Enterobacter* spp., and *P. aeruginosa*. Together, meropenem/vaborbactam is a novel combination that exhibits strong and specific activity against KPC-producing CRE. While Vaborbactam also possesses the capacity to inhibit ESBLs and AmpC β-lactamases, its supplementary activity is not necessary, as meropenem alone effectively stabilizes these β-lactamases. Though multi-agent treatments may benefit high-risk patients, mono-therapy may be enough for other patients. For instance, meropenem/vaborbactam alone showed higher cure rates and lower patient mortality and nephrotoxicity in individuals with predominantly bacteremic CRE infections compared to other drug combinations (49). Therefore, along with considering the type of carbapenemase, a successful treatment of CR-GNB infections also necessitates consideration of different infection types, the severity of the infection, susceptibility of the causative organism, and the patient’s general health condition. Thus, meropenem/vaborbactam is another viable option for KPC-producing CRE infections.

### 4.3 Imipenem/relebactam

Relebactam is a type of β-lactamase inhibitor, structurally similar to avibactam, that can inhibit common class A carbapenemases (e.g., KPC) and class C cephalosporinases (e.g., AmpC). *In vitro*, relebactam has been shown to reverse resistance to imipenem in KPC-producing *P. aeruginosa* but has no potentiation effect in isolates with class B or D carbapenemase activity (50). In an *in vivo C. elegans* model, imipenem/relebactam was found to be a significant treatment for KPC-producing *K. pneumoniae* infections (51). Furthermore, relebactam in combination with imipenem/cilastatin inhibited AmpC, thus restoring the susceptibility of *P. aeruginosa* to imipenem. The combination demonstrated better efficacy with lower mortality and nephrotoxicity for the treatment of patients infected with CR-GNB (52). It is worth noting that this study included high-risk patients with poor outcomes, and the combination of relebactam and IMI is a potential therapeutic option for ICU patients infected with CR-GNB. Against blaKPC-containing *P. aeruginosa*, Imipenem/relebactam was shown *in vitro* to have a higher inhibitory activity than meropenem/vaborbactam but lower than ceftazidime/avibactam (53). Therefore, rapid diagnosis of the carbapenemase genotype of CRE or CRPA is significant for β-lactam/β-lactamase inhibitor combinations (BL/BLI) selections in clinical settings.

### 4.4 Cefiderocol

The recently approved BL/BLIs expand the therapeutic options available for KPC-producing and OXA-48-producing Enterobacteriaceae. Cefiderocol, the first cephalosporin containing an iron-based carrier, has gained approval for the treatment of carbapenem-resistant non-fermenting bacteria, including *P. aeruginosa* and *A. baumannii* (54). Current studies have demonstrated significant *in vitro* activity and effectiveness of cefodilol against CR-GNB (55). Cefodilol exhibits inherent stability against a wide range of carbapenemases, including class A, B, and D, as well as class C cephalosporinase hydrolases. Patients infected with KPC, NDM, VIM, IMP, and OXA-48 harboring CRE experiencing BSI or urinary tract infections can potentially benefit from cefodilol therapy (56). However, it is important to acknowledge that resistance may arise when cefiderocol is employed in the treatment of CRE. Instances of cefiderocol resistance in these isolates can be attributed to factors such as the clinical environment, *in vitro* exposure to cefiderocol, or resistance to other β-lactam antibiotics (e.g., ceftazidime or cefepime) prior to treatment. The application of cefiderocol in these cases carries a risk of mutation resulting in the development of specific mutations, such as NDM-5 (57), KPC-41, KPC-50 (58), and OXA-427 (59).

A study by Falcone et al. (60) conducted in the ICU featured 10 patients with *A. baumannii* BSI and ventilator-acquired pneumonia. These individuals had previous treatment failures with antibiotics, including colistin, and had developed renal and hepatic injury. Clinical success and survival rates at 30 days were 70 and 90%, respectively, with cefiderocol treatment. Cefiderocol monotherapy for critically ill patients was revealed to result in a lower infection recurrence rate and higher clinical success compared to combination therapy using drugs like colistin (61). However, all-cause mortality was higher with cefiderocol monotherapy. This trend may be linked to the heightened risk of infection recurrence or death in critically ill patients, who commonly experience trauma-induced immune compromise, prolonged hospitalization, invasive procedures, and colonization of the skin by multidrug-resistant organisms. Consequently, in addition to the timely and accurate selection of appropriate drug therapy, implementing specific preventive and control measures against CR-GNB infections in the ICU setting is paramount.

### 4.5 Polymyxins

The newly approved BL-BLIs have emerged as the primary treatment options for CRE and CRPA infections. However, the treatment landscape for CRAB infections is becoming increasingly limited. Although BL-BLI therapy is recommended for urinary tract infections due to the high concentration of polymyxins in the urinary tract, it is still considered an alternative therapy for CRAB infections (62). According to the 2023 guidelines by the Infectious Diseases Society of America (IDSA) for the management of resistant Gram-negative bacteria infections, high-dose ampicillin-sulbactam in combination with other agents, including Polymyxin B, is recommended for the treatment of moderate-to-severe CRAB infections (63). Polymyxin B is specifically indicated for the treatment of severe infections, such as BSIs. However, the efficacy and safety of Polymyxin B as a monotherapy are not well-established. Thus, it is generally advised to administer Polymyxin B in combination with at least one other antimicrobial agent from a different class. Colistin, another approved drug from the polymyxin class of antibiotics, is considered a last-resort treatment for *A. baumannii* infections (64).

However, the emergence of polymyxin-resistant strains has been well-documented, potentially due to colistin’s prodrug nature and the prolonged presence of its active form in the body, which can predispose to resistance (65). Typically, colistin is recommended in combination with other agents for the treatment of CRE with CRPA infections. However, a clinical observational study (66) found that the difference in outcomes between patients treated with colistin sulfate alone versus in combination with other antibiotics was not statistically significant. The use of low-dose polymyxins in the treatment of multidrug-resistant *A. baumannii* infections may elevate the risk of mortality (66). A trial in 2018 demonstrated that colistin combined with meropenem treatment did not yield improved outcomes for severe infections caused by CR-GNB (67). Additionally, colistin therapy, especially when employed as part of combination therapy for patients with CR-GNB infections, may result in unfavorable clinical outcomes and potentially increase the risk of kidney injury in patients (68, 69). Combination therapy could heighten the probability of adverse effects, escalate the cost of antimicrobial therapy, and contribute to the development of antimicrobial resistance. Further clinical trials are imperative to establish the efficacy and safety of colistin as a complementary or alternative treatment for severe CR-GNB infections, particularly in cases where BL-BLI treatment proves ineffective.

### 4.6 Tigecycline and minocycline

Tigecycline is a novel intravenous antibiotic with broad-spectrum activity and is derived from minocycline. It has traditionally been considered the preferred treatment for infections caused by CRE. However, the latest guidelines from the IDSA recommend β-lactams as the primary option for treating CRE infections, with tigecycline as an alternate option if necessary (63). Combining tigecycline with colistin, carbapenems, or aminoglycosides is the most commonly used regimen for treating CRE infections. Studies comparing these combinations found that tigecycline-colistin was most effective against *Klebsiella*, while imipenem-colistin was best against *Escherichia coli* (70). Tigecycline combined with amikacin and colistin, or minocycline with cefoperazone-sulbactam, showed synergistic inhibitory activity against CRAB (71, 72). OXA-24-producing strains are more sensitive to tigecycline-amikacin and OXA-23-producing strains are more sensitive to tigecycline-mucin use [1]. Minocycline and tigecycline have lower nephrotoxicity compared to mucins versus aminoglycosides and can be used in combination with other drugs as another treatment option for CRAB (72). Notably, tigecycline-based regimens with high-doses (200 mg loading and 100 mg maintenance) showed lower mortality rates in ICU patients than standard doses (100 mg loading and 50 mg maintenance), and combination therapy with tigecycline was more effective than monotherapy (73). Consequent to exposure to tigecycline, resistance was induced in CRKp but tigecycline-resistant strains exhibited greater susceptibility to other drugs, including aminoglycosides, carbapenems, and cephalosporins (74). Sequential combination therapy with tigecycline and aminoglycosides may be a more effective approach to treating CRE.

### 4.7 Aminoglycosides

Aminoglycoside antibiotics possess strong bactericidal properties and remain effective in treating MDR-GNB. However, their application is somewhat limited due to the side effect of nephrotoxicity. Generally, aminoglycosides are not the primary treatment option for severe infections. However, they can still be considered as a therapeutic alternative for combating CR-GNB when other options are unavailable. This is usually done in combination with other drugs such as β-lactams (75). For instance, studies have shown that the combination of imipenem and amikacin has a synergistic effect on CR-GNB both *in vivo* and *in vitro* (76–78). Amikacin exhibits lower resistance than gentamicin in most CRE strains (79). Many studies have supported the use of aminoglycosides in the treatment of CRE infections in critically ill patients before the introduction of novel BL-BLIs (80). Furthermore, gentamicin has demonstrated the potential to reduce mortality in *K. pneumoniae* sepsis caused by class A β-lactamase-producing enzymes, including KPC-3, SHV-11, and TEM-1 (81). A recent case report highlighted successful treatment of a patient with a CRKp intracranial infection after craniotomy using intrathecal injection of gentamicin and intravenous injection of amikacin, which displayed gentamicin susceptibility (82).

Plazomicin, a next-generation aminoglycoside antibiotic, has demonstrated a lower minimum inhibitory concentration compared to other aminoglycosides, making it a potential treatment option for infections caused by carbapenemase-producing, NDM-producing CRE (83). In a multicenter, randomized, open-label phase III trial that compared plazomicin with colistin (both in combination with imipenem) for the treatment of severe infections in CRE, plazomicin proved to be effective with a relatively low mortality and complication rate (84). Due to the nephrotoxicity associated with aminoglycoside antibiotics, they are generally not used in combination with colistin. To ensure optimal efficacy and minimal toxicity, appropriate dosage, administration, and therapeutic drug monitoring of the patient are essential when using aminoglycosides.

## 5 Control strategies of CR-GNB

Infection control measures can be broadly classified into two types: horizontal and vertical strategies (85). Horizontal strategies are not pathogen-specific and aim to reduce infections caused by all pathogens. These strategies include standard precautions, such as hand hygiene, universal decolonization, and antimicrobial stewardship programs. On the other hand, vertical strategies are designed to target specific pathogens, involving carrier screening and contact precautions. The debate continues as to which of these two approaches is more effective. Nevertheless, implementing both strategies in parallel in the ICU setting may optimize infection control. Although monitoring the transmission route of CR-GNB is challenging, identifying high-risk groups is relatively simple. Implementing targeted prophylaxis and control measures for patients at high risk seems to be a promising approach. Furthermore, predictive or early warning models for CR-GNB infection are currently being explored and hold potential for application in the ICU.

### 5.1 Horizontal strategies

Hand hygiene: Multi-drug resistant organisms (MDROs) have exhibited the ability to persist in hospital environments, such as floors, walls, beds, doorknobs, bedside tables, and equipment (86). Barnes et al. (87) developed a patient–patient transmission model within the ICU and compared the effects of hand hygiene and environmental cleanliness on MDRO acquisition rates; findings suggest that universal decolonization methods could eliminate colonization of MDRO Gram-positive bacteria. For example, patients in the ICU receiving mupirocin nasal injection have lower rates of MRSA BSIs compared to those undergoing chlorhexidine bathing (88). Extensively resistant MDROs, such as CRE, have shown poor response to chlorhexidine treatment, and current clinical evidence does not support the removal of patient colonization (89, 90). A meta-analysis indicated that ICU bathing with chlorhexidine significantly reduces *A. baumannii* colonization (91). Compliance with hand hygiene is widely considered as the foundation for preventing MDRO spread in ICUs. However, in hospitals with low compliance rates, proactive detection of CR-GNB has substantial benefits for patients when implemented with increased environmental cleanliness. Nevertheless, in high hand hygiene compliance environments, contact precautions and screening for CR-GNB colonization contribute little to preventing MDRO spread, especially for CR-GNB.

Antimicrobial Stewardship: Antimicrobial Stewardship (AMS) is a significant measure of importance as defined by the IDSA. It entails implementing coordinated interventions aimed at enhancing and evaluating proper utilization of antimicrobials. This is accomplished through facilitating optimal selection of antimicrobial regimens, determining appropriate dosage, therapy duration, and administration route. For patients in the ICU, the potential negative consequences of antimicrobial overuse are considered less perilous compared to the inadequate employment of restraints. Reports indicate a substantial proportion of ICU patients receiving excessive antimicrobial therapy, which includes treatment involving antimicrobials for suspected infections, utilization of overly broad-spectrum antibiotics, delayed initiation of timely antibiotic de-escalation and optimization, and prolonged duration of therapy (92, 93). The implementation of Antibiotic Stewardship Programs (ASP) within the ICU setting can potentially reduce the misuse of antimicrobials, shorten hospital stays, minimize costs, and decrease the emergence of drug resistance (94). Also, the study conducted by Khdour et al. (95) highlights the importance of establishing a well-structured antimicrobial stewardship team in the context of AMS. They found that timely feedback and prospective audits from the antibiotic stewardship team, within 48–72 h of antibiotic administration for ICU patients, had a positive impact on patient outcomes. Calcitoninogen as a biomarker in the ICU has been shown to reduce the use of antibiotics and mortality rates to some extent (96). However, further investigation is needed to determine the efficacy of calcitonin as a treatment indicator, and the cost of frequent testing must be balanced with potential savings from shorter antibiotic therapy. To address the growing issue of carbapenem resistance, experts emphasize the importance of implementing clear strategies to guide the appropriate use of carbapenem antibiotics (97).

### 5.2 Vertical strategies

Rapid Diagnostic Tests: Standard microbial identification techniques typically take 48–72 h, while optimizing antibiotic therapy within the first 6–12 h of infection is critical for treating life-threatening infections. Rapid diagnostic tests (RDTs) provide assistance to ASP by contributing to timely and effective antimicrobial therapy, potentially reducing mortality, hospitalization, and costs, as well as improving antimicrobial use and clinical and economic outcomes. Recently developed RDTs are able to provide identification results within 3 h of collection and 2.5 h after Gram staining (98). The RDTs provide an opportunity to rapidly optimize antimicrobial therapy, but have been shown to be combined with ASP to maximize translation into improved patient outcomes (99). Studies have identified genotyping and phenotyping of *Escherichia coli*, *Klebsiella*, etc. based on RDTs to predict susceptibility to β-lactams (ceftazidime, piperacillin-tazobactam, imipenem, and meropenem). RDTs can support downgrading decisions for the treatment of GNB infections (99).

Screening and prophylactic isolation: Patients in the ICU are particularly vulnerable to colonization or infection with MDRO either upon admission or during their hospital stay due to various risk factors. To reduce the spread of MDRO, it is crucial to implement proactive screening or isolate patients with high-risk factors (100). Although proactive testing methods differ among hospitals in different regions, they usually involve obtaining fecal/rectal swabs from patients upon admission or at regular intervals (weekly or bi-weekly). This practice applies to all patients or those at high risk (e.g., ICU patients, those with a history of previous colonization/infection), with a focus on identifying CRE. Results of a study revealed a high incidence of CRKp colonization and a likelihood of eventual CRKp infection in patients who carried *Klebsiella pneumoniae* (including CRKp or carbapenem antibiotic-susceptible *Klebsiella pneumoniae*) upon ICU admission (101). Proactive screening in high-risk units for CRE colonization or infection has also shown that CRE-positive patients, both neonatal and non-neonatal, exhibit different genotypes of carbapenemases. Notably, over 90% of CRE-positive neonates carry NDM. Isolating and placing these patients appropriately may help reduce the risk of CRE infection (102).

Additionally, the implementation of proactive testing and isolation strategies has shown a decrease in infections caused by CRAB and CRPA at a broader scale (103). Hospitals with limited isolation facilities have commonly resorted to a contact precautions approach in confining ICU patients to their own beds, similar to horizontal measures. Notably, no transmission of resistant organisms, such as *Klebsiella pneumoniae*, was detected in these cases (89). Implementing universal contact precautions, regardless of the specific pathogen, may also impede the spread of CR-GNB within the ICU.

### 5.3 Predictive model and practical application

The emergence and spread of CR-GNB are influenced by various factors. Identifying high-risk factors to determine ideal target populations for proactive testing or prophylactic contact helps optimize the allocation of limited resources. Depending on the purpose of the prediction model, different target populations can be selected for retrospective or prospective studies. The CR-GNB infection prediction model and the early warning model in Table 2 were designed to identify patients infected with or carrying CR-GNB, respectively. In order to prevent the spread of CR-GNB before culture results are available, modeling techniques have been employed to assist in the pre-isolation of potential carriers of CR-GNB or patients who are at a high risk of infection upon admission. There is also a type of predictive modeling that predicts infection at a particular site. BSI is a severe infection characterized by positive blood cultures in patients displaying symptoms of systemic infection. BSI often leads to unfavorable outcomes for patients in the ICU, including longer hospital stays and higher mortality rates (109, 110). While blood cultures serve as the gold standard and primary tool for diagnosing pathogens causing BSI, they are susceptible to delays in initiating effective treatment due to the time required (111). In addition to performing timely blood cultures or rapid diagnostic tests when BSI is suspected, several studies have explored the use of predictive modeling to construct early warning models for BSI. Several studies, as depicted in Table 3, have developed early warning models to identify BSI in vulnerable populations, such as children, the older adults, and individuals with immunodeficiencies. These models rely on risk factors or biomarkers to target high-risk populations and implement prophylactic measures, thereby reducing the occurrence of BSI and the associated mortality risk. While most of these models have demonstrated reliable predictive performance, unfortunately, only a limited number of studies have conducted validation in diverse healthcare settings. Consequently, the geographical applicability of these models may be constrained due to this lack of validation across multiple centers.

**Table 2:** CR-GNB carriage or infection prediction models in ICUs.

Purpose of model application	Constructions and effects of the models	Factors for modeling	Methods	References
CR-GNB acquisition prediction in the ICU	CR-GNB culture-positive sample/culture-negative sizes is 343/1029. Model displays good result with an accuracy of ∼90% (no external validation).	(1) Increased Simplified Acute Physiology Score 3; (2) Severe chronic obstructive pulmonary disease; (3) Exposure to hemodialysis catheter; (4) Central venous catheter; (5) Mechanical ventilation.	Multiple logistic regression	(104)
CR-GNB carriage prediction in the ICU	CR-GNB culture-positive sample/culture-negative sample sizes of experimental and validation groups are 1385/1535 and 74/132. RF model is the optimal model; AUC of model are 0.91 (experimental cohort) and 0.92 (prospective validation cohort).	(1) Male sex; (2) Invasive catheterization; (3) Single room; (4) Mechanical ventilation; (5) Hospital residence history; (6) History of cephalosporins; (7) Systolic blood pressure; (8) Respiratory rate; (9) Glasgow Coma Scale; (10) APACHE II scores; (11) White blood cell count; (12) Hematocrit; (13) C-Reactive protein; (14) Direct bilirubin; (15) Total protein; (16) Fibrinogen	Multiple logistic regression; RF; XGBoost; Decision tree	(105)
CRO infection prediction in patients with the first ICU admission	CRO infection sample/total sample sizes is 183/4531. The effect is represented by the Nomogram; AUC is 0.723 (no external validation).	(1) Male sex; (2) Hemoglobin-min; (3) Temperature-max; (4) Use of a peripherally inserted central catheter line; (5) Dialysis treatment; (6) Use of carbapenems	Logistic regression	(106)
CR-GNB infection prediction in the ICU	CR-GNB infections sample/total sample size of experimental and validation groups are 143/205 and 69/104. The effect is represented by the Nomogram; AUC of model are 0.753 (experimental cohort) and 0.718 (validation cohort).	(1) Combination antibiotic treatments; (2) Hospital-acquired infection; (3) Mechanical ventilation ≥7 days	Multiple logistic regression	(107)
Identification of CR-GNB carriers during ICU admission	CR-GNB carries sample/total sample sizes is 183/1736. The effect is represented by the Nomogram; AUC is 0.83 (no external validation).	(1) Neurological disease; (2) High-risk department history; (3) Length of stay ≥14 days; (4) ICU history; (5) Invasive mechanical ventilation; (6) Gastrointestinal tube placement; (7) Carbapenem usage	Logistic regression	(108)

CR-GNB, carbapenem-resistant gram-negative bacteria; ICU, intensive care unit; APACHE II, acute physiology and chronic health evaluation; AUC, area under the curve; RF, random forest; XGBoost, extreme gradient boosting.

**Table 3:** BSI early warning models of critical patients.

Critical patients	Constructions and effects of the models	Methods	References
Febrile children with cancer	BSI sample/total sample sizes is 91/463; The effect is represented by probability score and nomogram; the C-index is 0.885 (no external validation).	Logistic regression	(112)
Severe/acute burn patients	BSI sample/total sample sizes are 118/222 and 59/272; The effects of the two models are represented by computing the formula and nomogram; the two models’ AUC are 0.84 (external validation) and 0.90 (no external validation).	Logistic regression	(113, 114)
CRE carriers in the ICU	BSI sample/total sample size is 21/42; The effect is represented by the probability score; AUC is 0.921 (no external validation).	Logistic regression	(115)
Patients using CVC	BSI sample/total sample size is 399/7468; AUC = 0.82 (no external validation).	RF; Forward selection; Lasso regression	(116)
Burned children	BSI sample/total sample size is 21/82; AUC = 0.938 (no external validation).	RF; Forward selection; Lasso regression	(117)
Pediatric cancer patients with HSCT	BSI sample/total sample size is 624/11183; AUC = 0.74 (no external validation).	ENR; SVM; XGBoost; GBM	(118)
Patients with suspected bacteremia	In an ICU and another ICU, BSI sample/total sample sizes are 151/2351 and 162/1021 respectively; The two models’ AUC are 0.89 and 0.92. The samples of the two centers are used for external verification of the models, and the outcomes are bad.	RF; XGBoost	(119)

The burn patients correspond to the early warning models for two categories of patients, severe burns and acute burns; the patients with suspected bacteremia in the ICU correspond to two models constructed from two samples from different centers. The rest are single-center BSI early warning models for such critical patients. BSI, bloodstream infection; ICU, intensive care unit; CVC, central venous catheter; HSCT, hematopoietic stem cell transplantation; AUC, area under the curve; RF, random forest; GBM, gradient boosting machines; SVM, support vector machine; XGBoost, extreme gradient boosting; ENR, elastic-net regression.

## 6 Discussion

The ICU is particularly susceptible to the emergence and spread of CR-GNB, necessitating the urgent strengthening and implementation of preventive measures within this high-risk setting. Currently, the range of antibiotics available for treating CR-GNB infections is limited. In the long term, it is crucial to prioritize the optimal utilization of existing antibiotics rather than relying solely on the development of new drugs. The presence of drug resistance genes in CR-GNB makes it difficult to promptly diagnose the pathogen and select suitable antibiotics. The high-density care provided in ICUs further increases the likelihood of cross-transmission of drug-resistant gene. This greatly affects the prognosis of ICU patients.

Having a thorough understanding of the common resistance genes found in CR-GNB and selecting appropriate antibiotics are crucial prerequisites for delaying the development of resistance. Pathogenic bacteria producing different genotypes of carbapenemases may have varying sensitivities to antibiotics. Selection of rational antibiotics based on enzyme genotypes not only controls the patient’s condition in time but also delays the development of drug resistance. Recent studies have highlighted the efficacy of newly approved BL-BLIs like ceftazidime/avibactam, meropenem/vaborbactam, and imipenem/relebactam as the first-line therapeutic options for most CRE and CRPA infections. However, these BL-BLIs have been found to be less effective in treating CRAB. For the treatment of CRAB and as an alternative when BL-BLIs are ineffective against KPC, NDM, VIM, IMP, and OXA-48 producing *Escherichia coli*, cefiderocol is recommended. While high-dose tigecycline has shown potential benefits in managing CR-GNB, conclusive evidence regarding its superiority over standard tigecycline dosing or the comparative effectiveness of combination therapy versus monotherapy remains elusive (120, 121). Monotherapy with cefiderocol has been shown to be more effective than combination therapy. Polymyxins, tigecycline, minocycline, and aminoglycosides are generally suggested as combination therapies or alternative treatments for CRE. Moreover, CR-GNB often exhibit a significant degree of co-resistance, limiting the range of effective therapeutic interventions. In cases where CR-GNB demonstrate resistance to key antibiotics such as fluoroquinolones, piperacillin, third-generation cephalosporins, and carbapenems, only colistin, aminoglycosides, tigecycline, fosfomycin, ceftazidime/avibactam, and ceftolozan/tazobactam are some of the few therapeutic options available (122).

The RDTs play a crucial role in ensuring that patients receive appropriate treatment in a timely manner, thereby decreasing the turnaround time for empirically prescribing broad-spectrum antibiotics. Additionally, RDTs aid in screening patients admitted to the ICU for carriage of CR-GNB, which is a vital preventive measure. The ICU requires strict infection control measures, such as hand hygiene, antimicrobial stewardship, proactive screening and prophylactic isolation, among other common practices. Another valuable tool for decision support is clinical predictive modeling, which can forecast the carriage and infection of drug-resistant bacteria. Currently, these models are typically built using multivariate logistic regression. However, the advancement of machine learning technology allows for the construction of infection-related models using large datasets and new algorithms, potentially improving their stability and effectiveness. The integration of machine learning algorithms with RDTs holds promise for enhancing the detection of predominant carbapenem resistance genes within clinical isolates of CR-GNB (123–125). This approach also enables the refinement of dosing regimens through the analysis of *in vitro* experimental data and pharmacodynamic considerations, thereby supporting the ASP of CR-GNB infections (126). It was discovered that most of the existing clinical prediction models based on machine learning for relevant infections lacked external validation, and those that were externally validated displayed poor performance. This aspect may also explain why prediction models for CR-GNB infection or carriage within the ICU are not widely implemented.

In order to improve patients’ prognosis and enhance their long-term quality of life, it is crucial to heighten vigilance against CR-GNB during ICU hospitalization. As well as administering antibiotics rationally based on the pathogen type and susceptibility, it is vital to swiftly identify the carbapenemase type in CR-GNB cases and take appropriate measures to prevent and control associated infections. We anticipate the emergence of more therapeutic strategies based on carbapenemase genotypes. We anticipate that future studies will delve further into treatment options based on genotypes of drug-resistant bacteria. Additionally, exploring CR-GNB-related models based on machine learning is expected to develop more effective infection control tools for ICU settings.

## Author contributions

QL: Writing – original draft, Writing – review & editing, Conceptualization, Investigation, Software. XZ: Investigation, Writing – review & editing. RY: Conceptualization, Investigation, Writing – review & editing. XS: Conceptualization, Investigation, Writing – review & editing. GL: Investigation, Software, Writing – review & editing. CZ: Conceptualization, Investigation, Writing – review & editing. PL: Investigation, Writing – original draft. SL: Conceptualization, Software, Writing – review & editing. JX: Investigation, Software, Writing – review & editing. YY: Conceptualization, Software, Writing – original draft, Writing – review & editing, Supervision.
